# Consistent condom use and associated factors among HIV positive women of reproductive age on anti-retroviral treatment in Ogun State, Nigeria

**DOI:** 10.11604/pamj.2022.43.101.32806

**Published:** 2022-10-25

**Authors:** Oluseye Ayodele Ajayi, Olabanjo Okunlola Ogunsola, Yewande Akinro, Scott Adamu-Oyegun, Kucheli Wudiri, Temitope Olumuyiwa Ojo, Babatunde Amoo

**Affiliations:** 1 Prevention and Community Service Directorate, APIN Public Health Initiatives, Federal Capital Territory, Abuja, Nigeria,; 2 Clinical Service Directorate, APIN Public Health Initiatives, Ibadan, Nigeria,; 3 Department of Community Health, Obafemi Awolowo University, Ile-Ife, Nigeria

**Keywords:** Condom use, human immunodeficiency virus, women living with HIV, Nigeria

## Abstract

**Introduction:**

consistent condom use remains an integral and essential part of comprehensive Human Immunodeficiency Virus (HIV) prevention and care programme. This study assessed consistent use of condom among HIV-positive women of reproductive age on antiretroviral treatment and its associated factors.

**Methods:**

a cross-sectional survey of 360 women living with HIV (WLHIV) receiving treatment in four APIN Public Health Initiatives, Nigeria supported President's Emergency Plan for AIDS Relief (PEPFAR) comprehensive Antiretroviral Therapy (ART) sites in Ogun State was conducted from October 2018 to March 2019. Information were collected on consistent condom use among respondents and their associated factors using a pre-tested questionnaire. Data were analysed using SPSS IBM version, with statistical significance set at 0.05.

**Results:**

the mean age of the women was 38.1 ± 5.8 years. About two-fifth (39.4%) of the respondents reported consistent use of condom. Of the 142 respondents who reported inconsistent use of condom, 51.6% and 37.5% mentioned sexual displeasure and partner’s disagreement respectively as reasons for inconsistent use. Factors associated with consistent condom use were marital status (p < 0.001), respondent’s occupation (p = 0.040), partner’s occupation (p = 0.012) and partner’s HIV status (p = 0.045). Respondents with HIV negative partners were 1.8 times more likely to use condom consistently compared to those with HIV positive partners (AOR = 1.826, CI = 1.018 – 3.274).

**Conclusion:**

this study concludes that there is poor consistent condom use among WLHIV in Ogun State. The rate is worse among the sero-concordant partners than the sero-discordant partners. More needs to be done through behaviour change communication aimed at improving consistent condom use among HIV clients.

## Introduction

It is estimated that 37.9 million people worldwide are currently infected with HIV, 36.2 million out of these are adults and 52% of whom are women [1]. In Nigeria, the population of people living with HIV in 2018 was estimated at 1.9 million [2] and 58% of them are women [3]. Most of whom are in the reproductive age group and with a higher risk for perinatal transmission of HIV; a major driver of new HIV infections globally [1]. Reports from the 2018 Nigerian HIV/AIDS Indicator and Impact Survey (NAIIS) showed that while Nigeria´s national HIV prevalence is 1.4% among adults aged 15-49 years, the prevalence among women aged 15-49 years indicates that they are more than two times likely to be living with HIV than men of the same age group (1.9% as against 0.9%) [4]. Women are disproportionately at risk of HIV/AIDs due to unequal cultural, social and economic status in society [5]. Various factors have been reported to make women more susceptible to contracting HIV namely; biological factors such as the anatomy of females and the task of child bearing. Other studies have identified other factors such as limited educational and employment opportunities, gender inequality, economic dependency, poor sexual negotiation, sexual violence, coercion, poverty and social norms [6].

Consistent condom use remains an integral and essential part of comprehensive HIV prevention and care programme. Condom has proven effectiveness in reducing risk of sexual transmission of HIV infection especially among HIV sero-discordant partners [7]. Results of Nigeria National Demographic Health Survey (2018) showed good knowledge of condom use among women of reproductive age in Nigeria. About 71% of women surveyed knew that consistent use of condoms and limiting sexual intercourse to one uninfected partner can reduce the risk of HIV [8]. In the 12 months before the survey, 9% of women reported having sexual intercourse with a person who neither was their husband nor lived with them, and fewer than 4 in 10 of those women (36%) used a condom during the last sexual intercourse with such partners. However, the most commonly used modern contraceptive by active unmarried women is the male condom (19%) with a considerably high awareness of male condom as a contraceptive method (76.6% amongst married women and 97.7% amongst sexually active unmarried women) compared to other family planning methods [8].

The indices were similar among sub-population of women living with HIV/AIDs (WLHIV). A study showed high level of awareness and knowledge about safe sex and condom use among WLHIV with 91.8% and 83.0% demonstrating good knowledge on the type and function of condom, and locations where condoms can be procured respectively [9]. Contrary to the high awareness, the rate of condom use among WLHIV in Nigeria is very low. The prevalence ranged from 29.4% in Abia, 38.4% in Port Harcourt to about 48.8% in Lagos [10-12]. Condom use was more prevalent among HIV sero-discordant partners compared to sero-concordant partners. Unlike the general population where factors such as level of education and location ranked as major influencers of condom use, factors shown to influence condom use among WLHIV were poor knowledge of the need for condom if both partners are HIV positive, having a regular sexual partner, difficulty negotiating condom use by women, assumption that their partners are already infected, desire to have children and the opinion that condom reduces sexual pleasure among others [13-15].

Antiretroviral Therapy (ART) has made it possible for HIV positive women to live healthier lives with better health status and with this comes a renewed interest in the resumption of sexual activities that could result in pregnancies most of which are unplanned as have been reported in various studies [16]. Such unplanned pregnancies are associated with consequences for the mother and the baby one of which is a high viral load that increases the risk of mother to child transmission. The first WHO Prevention of Mother to Child Transmissions (PMTCT) pillar is hinged on prevention methods, one of which is condom use. Globally, condom use is an essential strategy for preventing HIV transmission amongst any population including pregnant women and their partners. Condoms provide dual protection against unwanted pregnancies and sexually transmitted infections (STIs), the former being the key component of the second PMTCT Pillar for women living with HIV. When used correctly and consistently, condoms can reduce the risk of infection by 94% [17]. However, accessibility, availability, affordability, and correct use remain a challenge to maximizing the full potentials of condoms for STIs and pregnancy prevention [17]. This study aims to determine the proportion of HIV positive women of reproductive age who consistently use condom during sexual intercourse, the socio-demographic factors predicting consistent condom use and the association between condom use and their partners´ HIV status in Ogun State.

## Methods

**Study design:** a cross sectional study design that assessed consistent use of condom among HIV positive women of reproductive age on antiretroviral treatment in Ogun State, Nigeria.

**Setting:** Ogun State is one of the 36 States in Nigeria, located in South-Western part of the country. It has a population of 3,751,140 based on 2006 national census and an annual growth rate of 3.2%. Ogun State has twenty LGAs across the three senatorial districts in the State; Ogun Central, Ogun East and Ogun West senatorial districts [18]. According to the revised National HIV and AIDs strategic framework, Ogun State ranked as one of the HIV medium prevalence States in Nigeria with a prevalence of 1.6% [4]. The number of persons on treatment currently in the State as at 2018 was 12,100 and 52% are women of reproductive age. The State has about 1197 health facilities with full complement of HIV service being provided in 11 health facilities known as comprehensive HIV sites [19].

**Participants:** four hundred women of reproductive age were selected across four health facilities providing full complement of HIV services in Ogun State. The study population were women of reproductive age on HIV treatment and they must have been on treatment for at least one year. The study was done between June and December 2019 using a multistage sampling technique.

**Data sources/measurement:** interviews were done using a structured questionnaire that assessed consistent use of condom and its predictors among respondents, and the respondents´ partners´ HIV status. The questionnaire was adapted from the Nigeria National Demographic Health Survey (NDHS 2018) tool [8].

**Study size and sampling:** the sample size for the study was derived with the aid of Leshlie fisher´s formula for estimating single proportion; using a prevalence (29.8%) of consistent condom use from a past study [10], a 5% margin of error at 95% confidence limits, and 10% attrition rate, a final sample size of 360 was determined. The study was done in four out of six PEPFAR high volume comprehensive ART sites spread across the three senatorial districts in Ogun State. Two of the facilities were secondary health facilities while the other two were tertiary health facilities. High volume sites are sites with > 1000 PLHIV on treatment. The four facilities have an average patient load of sixty women of reproductive age per clinic day. In achieving a sample size of 100 per facility, every 6^th^ woman visiting the facilities per clinic day was interviewed over a data collection period of 10 clinic days.

**Statistical analysis:** data were cleaned and analysed using statistical package for social sciences (SPSS) version 23. The primary outcome variable was respondents´ consistent use of condom, elicited using three response categories; “always, often and sometimes”. The responses were dichotomized into ’Yes' or 'No', by making “always” response “Yes” and collapsing often and sometimes to “No”. The secondary outcome was partner HIV status, also dichotomized into positive and negative. Exploratory variables were respondent´s socio-demographic characteristics. Findings were presented using tables and charts and inferences were made using Chi-square test for categorical variable, t-test for continuous variable. Factors that were significant at bivariate level at p-value of less than 0.21 were introduced into multivariate logistic models to ascertain the predictors of respondent´s consistent condom use.

**Ethics:** ethical clearance for the study was obtained from Ogun State Ministry of Health Ethics Committee. Written consents were obtained from respondents before the interviews.

## Results

Three hundred and sixty (360) women of reproductive living with HIV were interviewed during their routine HIV clinics. The mean age of the women was 38.1 ± 5.8 years, with mean parity of 3.0 ± 1.0. Majority of the respondents were married 293 (81.4%), business women 247 (70.2%), living in a monogamous setting 195 (63.7%). A higher percentage had secondary education 200 (55.6%). About half of the respondents´ partners had secondary education 172 (47.8%) and HIV sero-negative 187 (56.2%) Table 1. Slightly above one-third (39.4%) of the interviewed respondents reported consistent use of condom during sexual intimacy %). In addition, 51.6% and 37.5% of the 142 respondents who reported inconsistent use of condom mentioned sexual displeasure and partner´s disagreement respectively as the reasons for inconsistent use Table 2. Factors that were statistically significantly associated with consistent use of condom were respondent´s marital status (p < 0.001), respondent´s occupation (p = 0.040), respondent´s partner occupation (p = 0.012) and partner´s HIV status (p = 0.045). Lower percentage of respondents who were separated/divorced 7 (18.4%) or widowed 2 (9.5%) reported consistent use of condom compared to their counterpart who were married 130 (44.4%) or single 3 (37.5%). Furthermore, a lower percentage of respondents whose partners were HIV positive, 30 (32.6%) reported consistent condom use when compared to those whose partners were negative 84 (45.2%) Table 3. Respondents who were artisans, had lower likelihood of being consistent with condom use when compared with those that were public/civil servants (Adjusted OR = 0.284, CI = 0.091 - 0.882, P = 0.030). Furthermore, respondents with HIV negative partners were about twice more likely to be consistent with condom use compared to those with positive partners (Adjusted OR = 1.826, CI = 1.018 - 3.274, P = 0.043) Table 4.

**Table 1:** respondents’ background characteristics

Variable (s)	Frequency	Percentage (%)	Mean ± S.D
**Age group (completed years) n=360**			
20-29	24	6.7	38.1 ± 5.8
30-39	182	50.6	
40-49	154	42.8	
**Parity (n=326)**			
< 4	247	75.8	
4 and above	79	24.2	
**Marital status (n=360)**			
Married	293	81.4	
Single	8	2.2	
Separated/divorced	38	10.6	
Widow	21	5.8	
**Educational attainment (n=360)**			
No formal education	12	3.3	
Primary	77	21.4	
Secondary	200	55.6	
Tertiary	71	19.7	
**Occupation (n=352)**			
Public/civil servant	52	14.8	
Artisan	36	10.2	
Business/farming	247	70.2	
Student	2	0.6	
Unemployed/housewives	15	4.3	
**Family setting (n=306)**			
Polygamous	111	36.3	
Monogamous	195	63.7	
**Partner's educational level (n=360)**			
No formal education	5	1.4	
Primary	34	9.4	
Secondary	172	47.8	
Tertiary	99	27.5	
Don't know	50	13.9	
**Partner's occupation (n=360)**			
Public/civil servant	94	26.1	
Artisan	96	26.7	
Business/farming	133	36.9	
Student/unemployed	2	0.6	
Don't know	35	9.7	
**Partner's HIV status (n=333)**			
Positive	92	27.6	
Negative	187	56.2	
Unknown	54	16.2	

**Table 2:** condom use among HIV-positive women of reproductive age

Variable	Frequency	Percent
**Consistent use of condom**		
No	218	60.6
Yes	142	39.4
**Total**	**360**	**100.0**
**Main reason for inconsistent condom use**		
Partner disagrees with me	30	22.6%
Sexual displeasure	75	56.4%
Never seen no heard any information before on condom	6	4.5%
Previous use failed/fear of side effects	22	16.5%
**Total**	**142**	**100.0**

**Table 3:** association between respondents’ socio-demographic factors and consistent condom use

Variable (s)	No (n = 218)	Yes (n = 142)	Total	Statistical indices
	Freq (%)	Freq (%)		
**Age group (in completed years)**				ꭕ = 3.18, df = 2, p = 0.203
20-29	11 (45.8)	13 (54.2)	24	
30-39	108 (59.3)	74 (40.7)	182	
40-49	99 (64.3)	55 (35.7)	154	
**Parity**				ꭕ = 0.54, df = 1, p = 0.462
< 4	148 (59.9)	99 (40.1)	247	
4 and above	51 (64.6)	28 (35.4)	79	
**Marital status**				LR = 20.34, df = 3, p < 0.01**
Single	5 (62.5)	3 (37.5)	8	
Married	163 (55.6)	130 (44.4)	293	
Separated/divorced	31 (81.6)	7 (18.4)	38	
Widow/widower	19 (90.5)	2 (9.5)	21	
**Educational level**				ꭕ = 5.49, df = 3, p = 0.14
No formal education	5 (41.7)	7 (58.3)	12	
Primary	53 (68.8)	24 (31.2)	77	
Secondary	122 (61.0)	78 (39.0)	200	
Tertiary	38 (53.5)	33 (46.5)	71	
**Occupation (n= 213; 139)**				LR = 10.14, df = 4, p = 0.04**
Public/civil servant	25 (48.1)	27 (51.9)	52	
Artisan	25 (69.4)	11 (30.6)	36	
Business/farming	156 (63.2)	91 (36.8)	247	
Student	0 (0.0)	2 (100.0)	2	
Unemployed/housewives	7 (46.7)	8 (53.3)	15	
**Family setting (n = 176; 130)**				ꭕ = 0.04, df = 1, p = 0.84
Polygamous	63(56.8)	48 (43.2)	111	
Monogamous	113 (57.9)	82 (42.1)	195	
**Partners level of education (n=311)**				LR = 1.48, df = 3, p = 0.690
No formal education	2 (40.0)	3 (60.0)	5	
Primary	22 (64.7)	12(35.3)	34	
Secondary	97 (56.4)	75 (43.6)	172	
Tertiary	58 (58.6)	41 (41.4)	99	
**Partner's occupation (n = 315)**				LR = 12.94, df = 4, p = 0.012**
Public/civil servant	51 (54.3)	43 (45.7)	94	
Artisan	43 (44.8)	53 (55.2)	96	
Business/farming	88 (66.2)	45 (33.8)	133	
Student	1 (100.0)	0 (0.0)	1	
Unemployed	1 (100.0)	0 (0.0)	1	
**Partner HIV status (n = 278)**				ꭕ = 4.00, df = 1, p = 0.045**
Negative	102 (54.8)	84 (45.2)	186	
Positive	62 (67.4)	30 (32.6)	92	

** significant at p<0.05

**Table 4:** predictors of consistent condom use

Variable (s)	Adjusted OR	95% C.I. for EXP(B)		Sig.
		Lower	Upper	
**Age group (in completed years)**				
20-29	Ref			0.825
30-39	1.231	0.371	4.077	0.734
40-49	1.038	0.299	3.598	0.953
**Parity**				
< 4	Ref.			
4 and above	0.611	0.314	1.190	0.147
**Marital status**				
Widow/widower	Ref			0.398
Single	0.000	0.000		0.999
Married	6.436	0.685	60.495	0.103
Separated/divorced	4.464	0.346	57.510	0.251
**Occupation**				
Public servant	Ref			0.203
Artisan	0.284	0.091	0.882	0.030**
Business/farming	0.602	0.285	1.273	0.184
Student	0.000	0.000		1.000
Unemployed/housewife	1.242	0.292	5.282	0.769
**Partner's occupation**				
Public servant	Ref			0.123
Artisan	1.766	0.844	3.695	0.131
Business/farming	0.674	0.343	1.322	0.251
Student	0.000	0.000		1.000
Unemployed	0.000	0.000		1.000
**Partner's HIV status**				
Positive	Ref			
Negative	1.826	1.018	3.274	0.043**

** significant at p<0.05, Ref=Reference category

## Discussion

This study assessed the factors associated with consistent condom use among WLHIV attending selected ART clinics in Ogun State. Condom use is a critical part of a comprehensive and sustainable approach to the prevention of HIV and other sexually transmitted infections (STIs) and for preventing unplanned pregnancies especially among PLWHA. The greatest protective effect of condoms is attained when they are used correctly and consistently rather than occasionally.

Findings from this study suggest that the prevalence of consistent condom use among HIV positive women of reproductive age group on ART is low in Ogun State with 39.4% reporting consistent use. This result draws attention to the need to emphasize adherence to preventive interventions during routine clinic visits among these group of people. Similar studies on condom use among PLHIV conducted in Nigeria showed low level of consistent use (29.4% in Abia, 38.4% in Port Harcourt and 48.8% in Lagos) [10-12]. While the studies in Lagos and Port Harcourt had both males and females as respondents, the study in Abia had only female PLHIV as respondents like our study. The findings from our study also compares with studies done in other Africa countries such as Malawi and Tanzania which also showed a similarly low level of consistent condom use (38.2% and 16.1% respectively) [13,20]. High rates of consistent condom use (71.3% and 78.9%) were reported in two studies in Brazil and Ethiopia respectively [21,22]. In both studies, the male population contributed more to the higher rate of consistent condom use more than their female counterparts. This perhaps suggests that there are still some gender and cultural factors that affect condom use. According to UNAIDS condom statement, women and young girls are usually and continually denied information and access to condoms. In addition, they are unable to negotiate the use of condoms during sexual intercourse. This needs to be taken into consideration when designing condom promotion programmes [23].

Major contributors to inconsistent condom use in this study were sexual displeasure and partner´s disagreement. These factors were described in a similar study among PLHIV on ART in Lagos State, Nigeria where 52.5% and 27.1% of the female interviewed posited that reduced sexual pleasure and partner refusal respectively were the major reasons for their inconsistent condom use [11]. A similar study in Abia, Nigeria also reported husband dislike as a major reason for non-use of condom among women living with HIV [10]. Other factors recognized to influence inconsistent condom use across these studies include key population, poor knowledge of the need for condom if both partners are HIV positive, having a regular sexual partner, difficulty negotiating condom use by women, assumption that their partners are already infected, desire to have children and the opinion that condom reduces sexual pleasure among others [13-15].

An important factor that was associated with consistent condom use in this study was the marital status of respondents. Single and married women reported a higher rate of consistent condom use than their widowed and separated counterparts. This is similar to findings from a study conducted in Ethiopia where divorced respondents were about five times less likely to use condom consistently as compared with single women [24]. This may be partly explained by the fact that widowed women are more likely to have fewer sexual exposures than their married or single counterparts. However, contrary to this finding, marital status did not appear to be associated with consistent condom use in other reviewed studies conducted in similar settings in Malawi, Tanzania and Ethiopia [13,20,25,26]. Furthermore, our study showed significant association between respondent´s partner occupation and respondent´s partner HIV status. Other socio-demographic characteristics assessed in our study which included age, parity, marital status, educational attainment, occupation, and family setting was not associated with consistent condom use. Although other studies done in similar settings in Africa have reported that participants with higher educational status are more likely to use condoms consistently compared to those with lower educational status, our study did not show any significant association between educational status and consistent condom use [25-27].

Our study showed a higher likelihood of consistent condom use among respondents whose partners were HIV negative when compared to those whose partners were HIV positive. This should be expected as consistent condom use is supposed to reduce the chances of HIV transmission, hence some PLHIV may embrace consistent use of condoms since they may not want their partners to get infected. This finding compares with a study done among group of MSM in Nigeria which showed partners HIV positivity as a negative predictor of consistent condom use [28]. Surprisingly, this finding contrasts with the findings from the studies in Malawi, Ethiopia, and Brazil where there was no significant association between consistent condom use and partner´s HIV status [13,21,25]. The difference is probably due to the different definitions of consistent condom use across the studies. Our finding is however consistent with the study in Tanzania where there was a significant association between consistent condom use and partner´s HIV positive status.

The strength of the study is that it focuses on an important aspect of public health that can considerably reduce new HIV infections, re-infection, and treatment failure if interventions to improve consistent condom use are in place. Some of the study limitations include the use of self-reported information to assess condom use, which may be subject to recall or socially desirable response bias. Earlier studies have shown that self-reported condom use may not be a reliable indicator of true condom use as it may be subject to significant reporting bias. In addition, this study was conducted in high volume ART clinics only and those who do not have scheduled clinic visits during the data collection period were excluded from the study; this could introduce some selection bias thus limiting generalizability. Future studies using qualitative or mixed methods approaches are required to assess social and cultural factors that may underline inconsistent use of condoms among WLHIV. Also studies with large sample sizes may be indicated to assess self-efficacy of condom use and the wider determinants of consistent condom use among this subgroup of WLHIV.

## Conclusion

This study concludes that there is a low rate of consistent condom use among women of reproductive age living with HIV in Ogun States. The rate is worse among the sero-concordant partners than the sero-discordant partners. Major contributors to the low rate were decreased sexual pleasure and partner´s disagreement. This suggests that more needs to be done through behaviour change communication with the aim of improving consistent condom use among HIV clients. Social marketing strategies targeted at overcoming major barriers to condom use among PLHIVs such as sexual displeasure and partner´s disagreement, should also be encouraged. Widows and separated/divorced women had very low rates of consistent condom use in this study, hence condom promotion needs to be intensified among this sub group of WLHIV.

### What is known about this topic

- Condom has proven effectiveness in reducing risk of sexual transmission of HIV infection;
- Not all WLHIV in the reproductive age group use condom consistently in Nigeria.

### What this study adds

- This study confirms the low rates of consistent condom use among WLHIV in the reproductive age group in Ogun State, Nigeria;
- Women living with HIV (WLHIV) who are artisans were less likely to use condom consistently while those with HIV negative partners were more likely to use condom consistently.

## References

[1] World Health Organization (2018). Global Health Observatory (GHO) data: HIV/AIDS.

[2] Federal Republic of Nigeria (2017). National HIV and AIDS strategic plan 2017-2021.

[3] UNAIDS (2016). Prevention gap report.

[4] Federal Ministry of Health (2019). Nigeria HIV/AIDS Indicator and Impact Survey (NAIIS) - South West zone summary fact sheet.

[5] Amin A (2015). Addressing gender inequalities to improve the sexual and reproductive health and wellbeing of women living with HIV. J Int AIDS Soc.

[6] World Health Organization (2017). Consolidated guideline on sexual and reproductive health and rights of women living with HIV- guidelines.

[7] UNFPA Position statement on condoms and HIV prevention, July 2004.

[8] National Population Commission (NPC) [Nigeria] and ICF (2019). Nigeria demographic health survey 201.

[9] Salaudeen AG, Musa OI, Ojotule A, Yusuf AS, Durowade KA, Omokanye LO (2014). Condom use among people living with HIV/AIDS attending Abejukolo General Hospital in Kogi State, North Central Nigeria. Ann Afr Med.

[10] Nduka I, Enwereji EE, Nduka EC, Ahuizi ER (2014). Determinants of consistent condom use among HIV-positive women in Abia State, South-East Nigeria. Journal of Clinical Research in HIV/AIDS and Prevention.

[11] Ayoola OD, Victoria GO, Bamidele O, Olufela KO, Oluwatosin SE, Mbaneifo EP (2014). Pattern, challenges and correlates of condom use among Nigerians living with HIV infection. Asian Pacific Journal of Tropical Biomedicine.

[12] Efobi CC, Okoye HC, Omunakwe H, Onodingene MN (2021). Condom use amongst people living with HIV/AIDS on antiretroviral therapy in Port Harcourt Nigeria. The Nigerian Health Journal.

[13] Haddad LB, Tang JH, Krashin J, Ng’ambi W, Tweya H, Samala B (2018). Factors associated with condom use among men and women living with HIV in Lilongwe, Malawi: a cross-sectional study. BMJ sexual & reproductive health.

[14] Deuba K, Kohlbrenner V, Koirala S, Ekström AM, CAT-S group (2018). Condom use behaviour among people living with HIV: a seven-country community-based participatory research in the Asia-Pacific region. Sex Transm Infect.

[15] Gursahaney PR, Cordes S, Ofotokun I, Wall KM, Jamieson DJ, Haddad LB (2019). Factors associated with condom use among HIV-positive women living in Atlanta, Georgia. PloS one.

[16] Sule H, Gyang M, Oyebode T, Tersoo M (2020). Contraception choice among HIV-positive women utilizing family planning services integrated with HIV care at the antiretroviral therapy clinic of Jos University Teaching Hospital, Nigeria. Eur J Med Heal Sci.

[17] Federal Ministry of Health (Nigeria) (2016). National guidelines for HIV prevention, treatment and care.

[18] Wikipedia Ogun State.

[19] Federal Ministry of Health Nigeria Nigeria health facility registry.

[20] Conserve D, Sevilla L, Younge S, Mbwambo J, King G (2012). Condom use among HIV-positive sexually active adults and partner´s HIV status in Dar es Salaam, Tanzania. J Health Care Poor Underserved.

[21] Lotfi R, Salehifar D, Jahromi AG (2020). Predictors of condom use among people living with HIV in Karaj: a cross-sectional study. Shiraz E Med J.

[22] Reis RK, Melo ES, Gir E (2016). Factors associated with inconsistent condom use among people living with HIV/Aids. Rev Bras Enferm.

[23] UNAIDS UNFPA WHO UNAIDS (2015). Position statement on condoms and the prevention of HIV, other sexually transmitted infections and unintended pregnancy.

[24] Ali MS, Tesfaye-Tegegne E, Kassa M, Tesfaye-Tegegne K (2019). Consistent condom use and associated factors among HIV-positive clients on antiretroviral therapy in North West Ethiopian Health Center, 2016 GC. AIDS Res Treat.

[25] Shewamene Z, Legesse B, Tsega B, Bhagavathula AS, Endale A (2015). Consistent condom use in HIV/AIDS patients receiving antiretroviral therapy in northwestern ethiopia: Implication to reduce transmission and multiple infections. HIV AIDS (Auckl).

[26] Dessie Y, Gerbaba M, Bedru A, Davey G (2011). Risky sexual practices and related factors among ART attendees in Addis Ababa Public Hospitals, Ethiopia: a cross-sectional study. BMC Public Health.

[27] Macharia AG, Kombe Y, Mwaniki P (2015). Consistent condom use among HIV positive women attending comprehensive care centre of Thika level 5 hospital, Kenya. World J.

[28] Olawore O, Crowell TA, Ketende SC, Ramadhani HO, Liu H, Ake JA (2021). Individual and partnership characteristics associated with consistent condom use in a cohort of cisgender men who have sex with men and transgender women in Nigeria. BMC Public Health.

